# CamNuvem: A Robbery Dataset for Video Anomaly Detection

**DOI:** 10.3390/s222410016

**Published:** 2022-12-19

**Authors:** Davi D. de Paula, Denis H. P. Salvadeo, Darlan M. N. de Araujo

**Affiliations:** IGCE—Institute of Geosciences and Exact Sciences, UNESP—São Paulo State University, Rio Claro 13506-900, SP, Brazil

**Keywords:** video anomaly detection, dataset, human behaviour analysis, weakly supervised, activity recognition, video surveillance, deep learning

## Abstract

(1) Background: The research area of video surveillance anomaly detection aims to automatically detect the moment when a video surveillance camera captures something that does not fit the normal pattern. This is a difficult task, but it is important to automate, improve, and lower the cost of the detection of crimes and other accidents. The UCF–Crime dataset is currently the most realistic crime dataset, and it contains hundreds of videos distributed in several categories; it includes a robbery category, which contains videos of people stealing material goods using violence, but this category only includes a few videos. (2) Methods: This work focuses only on the robbery category, presenting a new weakly labelled dataset that contains 486 new real–world robbery surveillance videos acquired from public sources. (3) Results: We have modified and applied three state–of–the–art video surveillance anomaly detection methods to create a benchmark for future studies. We showed that in the best scenario, taking into account only the anomaly videos in our dataset, the best method achieved an AUC of 66.35%. When all anomaly and normal videos were taken into account, the best method achieved an AUC of 88.75%. (4) Conclusion: This result shows that there is a huge research opportunity to create new methods and approaches that can improve robbery detection in video surveillance.

## 1. Introduction

The research area of video surveillance anomaly detection is a promising visual understanding area with several real applications. The goal is to learn the normal pattern from surveillance videos and detect situations that deviate from normality [1,2,3,4,5,6,7,8]. These situations are known as abnormalities or anomalies, and they may include car crashes, fights, robberies, violence, thefts, explosions, etc. An anomaly can be defined as a rare event that occurs in the video, but the exact appearance of the anomaly and how rare it needs to be depend on the context and can change according to different interpretations. Note that the variety of anomalies increases the difficulty of designing a system that performs well in all environments. Although approaches in the action recognition research area are well developed [9,10], they cannot be applied directly to video surveillance anomaly detection since the actions that could be considered anomalies (theft, violence, assault, etc.) may have a large temporal window, present huge differences in actions of the same nature (e.g., assault) and may involve several people and iterations, resulting in the poor efficiency of state–of–the–art methods [1,2,3].

A system that detects one or more situations that are considered abnormal could be designed using supervised deep learning, but the main difficulty in this case is the need for a huge dataset, since the occurrence of such events in this context is rare in the video data. Furthermore, even if a big dataset was available, the labelling process is expensive, since the annotations have to be made at the frame level to indicate exactly where the abnormality starts and ends in each video.

To deal with these problems, a classical approach is used to consider the anomaly detection process as a reconstruction problem [11,12,13]. Considering that normal videos are easier to obtain, these methods learn the probability distribution of normal videos by trying to reconstruct them. Since only normal videos are used to train a reconstructor, the idea is that when a video containing an abnormality is presented to this reconstructor, an error greater than a given threshold will appear, indicating an anomalous event. Following this framework, over the past few years, many works have tried to give special attention to different elements in a video, aiming to improve the abnormality detection quality. Some classical methods aimed to detect an anomaly through human tracking [14], but this approach provides poor performance in the real world due to the variety of occlusions in each scene [15]. Other approaches used flow estimation (one frame is subtracted from the other, and the result represents the motion between them) to extract features from frames [7,13,16].

However, these methods provide poor performance for real–world videos. Thus, aiming to improve real–world anomaly detection, Sultani et al. [1] proposed a dataset called UCF–Crime, which is composed of 1900 real–world video clips, and a method that uses multiple instance learning to find samples with abnormalities for the training process. This method decreases the cost of labelling, since the dataset only has to be weakly labelled; i.e., each video only needs to be binary–annotated (video–level annotation) to specify whether or not an anomaly exists in the video. There is no need for frame–level annotation.

Thereafter, several publications made progress in real–world anomaly detection, but because UCF–Crime has 13 anomaly categories, each type of anomaly has a few video clips. Taking into account that human–related crimes (robbery, burglary, assault, etc.) represent a problem that must be alleviated, this paper focuses on the robbery anomaly and proposes a new dataset for this type of crime. Thus, the main contributions of this work are as follows:

- A dataset containing 486 new real–world video surveillance clips showing robberies that were acquired from public sources such as social media is presented.
- A benchmark is created through the application of three state–of–the–art video surveillance methods that were originally applied to the UCF–Crime dataset. These methods were standardised for a fair comparison, which was not performed in the original UCF–Crime comparison.

The remainder of this text is organised as follows: In Section 2, related studies are presented. In Section 3, details and statistics concerning the new dataset presented in this work are described. In Section 4, results from the benchmark realised using this new dataset are discussed. In Section 5, the conclusions of this work are presented.

## 2. Related Work

Optimisation based on the reconstruction error is widely used in video surveillance anomaly detection because only normal videos are needed in the training phase and there is no need to label these normal videos. Several approaches belong in this paradigm:

- Classical Methods: In this category are the non–deep–learning–based methods, such as that proposed in [17], which focused on capturing pedestrian movement information using a histogram of oriented gradients (HOG) [18], object positions, and colour histograms. Khan et al. [19] used the Lukas–Kanade method [20] to calculate the flow direction from a pixel’s spatial vicinity (in the feature extraction step) and used k–means to determine an abnormality score for a video segment. This work focused on low–power devices and presented interesting results, taking into account the low–power needs of these devices. Amraee et al. [21] used the histogram of optical flow (HOF) [22] to represent the fixed part of an image (background) and the HOG [18] and local binary pattern (LBP) [23] to extract the features of moving objects from images (foreground).
- Hybrid Methods: These methods aim to model some part of the anomaly detection using classical models and deep learning. Rezaei and Yazdi [24] used a convolutional neural network (CNN) [25] and non–negative matrix factorisation to extract features from pixels. Then, they used a support vector machine (SVM) [26] to classify the features according to whether they are from a normal or abnormal segment. The method proposed in [7] used the Canny edge detector [27] to extract the features from the flow of a frame set. These features and the original images are combined and used as the input of a deep autoencoder, which was trained in an unsupervised manner. Wang et al. [28] used the Horn–Schunck [29] optical flow descriptor to extract the frame information and a deep autoencoder to calculate the reconstruction error.
- Deep Learning Methods: These approaches are based only on deep learning. Singh and Pankajakshan [11] used a CNN to extract local visual features (in the same frame) and long short–term memory (LSTM) [30] to aggregate features from several frames. Then, deconvolution [31] is used to reconstruct a future frame, which, in the training phase, is compared with the real future frame. In the test phase, an abnormality is detected if a frame has a reconstruction error greater than a predetermined threshold. Wang et al. [32] replaced the deconvolution part with a generative adversarial network (GAN) [33] that was used to predict a future frame given a set of the previous frames. They used PSNR as an error measure and the optical loss, intensity loss, and gradient loss to capture specific characteristics of the images. Following this GAN scheme, Doshi and Ylimaz [12] proposed a statistical approach to automatically configure the reconstruction error threshold that is used to detect abnormal video segments, since this is an important design choice.

Since the reconstruction error approach cannot provide good results on real–world datasets [1,2,3], Sultani et al. [1] proposed the multiple instance learning (MIL) approach for video surveillance anomaly detection, which does not require accurate frame–level annotation. In this approach, the exact localisation of an anomaly in a video is unknown. Instead, only video–level labels indicating whether or not the whole video contains some anomaly are needed. In the training phase, this method begins by dividing the input videos into a fixed number of segments. In each epoch, the segments of a positive video, i.e., a video that contains at least 1 anomalous segment, and a negative video, i.e., a video that contains no anomalous segments, are used to train a deep neural network using the proposed MIL ranking loss. In the remainder of this work, the method proposed by Sultani et al. [1] will be referred to as RADS (real–world anomaly detection surveillance). Sultani et al. [1] also presented a dataset containing weakly labelled videos with 13 types of realistic anomalies, such as fighting, road accidents, and burglary.

In [2], the robust temporal feature magnitude (RTFM) learning method was proposed; instead of using only one segment in each video to perform a training step in the MIL approach, it ranked *k* segments from each video. Furthermore, this method utilised an attention mechanism called non–local neural networks [34] to aggregate visual information in different frames (separated in time). To capture information from a single frame, the method used the pyramid of dilated convolution [35]. Other works have explored different architectures using the MIL approach. For example, weakly supervised anomaly localisation (WSAL) was used by Tian et al. [2]; they used high–order context encoding (HCE) for frame aggregation and feature extraction.

Other datasets were proposed in the literature to allow the development of video surveillance anomaly detection methods, but these datasets are limited in terms of the camera variation, resolution, point of view, weather conditions, etc. They offer alternative benchmarks for current academic works on video surveillance anomaly detection. The UCSD dataset [36] contains videos with a few types of anomalies, such as people walking in unusual paths and locations. The Avenue dataset [37] presents videos from the CUHK campus avenue. The Boss dataset [38] includes videos and audio from multiple cameras of people’s behaviour inside a moving train. The Subway Entrance and Subway Exit datasets [39] contain videos from, respectively, the entrance and exit of a subway station. Table 1 presents a comparison of several datasets in terms of the video quantity, average frame number, and dataset abnormality types.

## 3. Materials and Methods

In Section 3.1, the multiple instance learning approach is described. In Section 3.2, the CamNuvem dataset is discussed; it is also compared with other existing datasets to demonstrate its relevance. Section 3.3 describes how the benchmark is designed and what feature extractor was used. Section 3.4, Section 3.5 and Section 3.6 present a detailed description of the approaches used to evaluate the CamNuvem dataset; these approaches are robust temporal feature magnitude learning (RTFM) [2], weakly supervised anomaly localisation (WSAL) [2], and real–world anomaly detection in surveillance (RADS) [1], respectively. These methods were chosen for the initial CamNuvem dataset benchmark for the following reasons: RTFM and WSAL are current state–of–the–art video surveillance anomaly detection methods that use the multiple instance learning approach, and RADS is a classical method that was first introduced as an initial benchmark for the UCF–Crime dataset. In Section 3.7, the obtained results are discussed.

### 3.1. Multiple Instance Learning (MIL) Approach

Each video is called a bag and is divided into several segments. In the training phase, a deep neural network (DNN)–based model receives the following two input videos: (i) a video containing at least 1 anomaly segment, and (ii) a video containing no anomaly segments. The output of the DNN is an abnormality score for each segment of the two bags (videos). The objective function aims to increase the distance from the higher–scoring segment in the positive bag to the higher–scoring segment in the negative bag, decreasing the number of false negatives and increasing the number of true positives. The videos need to be weakly labelled, which requires much less effort than normal labelling. Thus, this approach uses information from abnormal videos in the training process, unlike the reconstruction error approach, providing an increase in the performance [1].

### 3.2. CamNuvem Dataset Statistics

The CamNuvem dataset consists of 972 videos in total, including 486 abnormal videos collected from public sources such as YouTube, Facebook, and other social media websites. The videos were edited to remove useless segments that were not recorded by the surveillance camera, such as introductions and endings. All the abnormal videos consist of people committing robberies. This category of videos was split into 437 videos for the training set and 49 videos (10%) for the test set. As with UCF–Crime [1], this dataset is weakly labelled, which means that only video–level labels are available for the training videos and frame–level labels are provided for the test videos. The annotators marked a video segment as abnormal only if a robbery was explicitly happening. If a hypothetical camera captures people committing a robbery, and then these people exit the camera view field and return moments later, there will be two abnormal segments with a normal segment between them. The decision to make a segment abnormal does not depend on past events. This feature can be inserted in future datasets and explored in future studies.

There are also 486 normal videos extracted randomly from UCF–Crime; 437 of these videos were used for the training set and 49 were used in the test set. Thus, the main novelty of this dataset is the aggregation of a new robust set of abnormal videos to improve the development of robbery detectors, which can be used with datasets that are already available.

**Figure 1:**
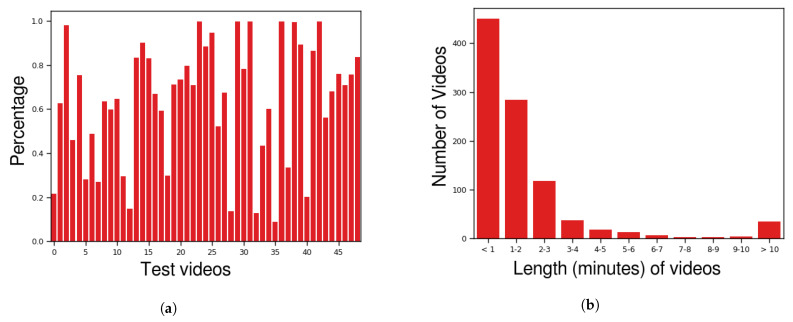
CamNuvem dataset statistics. (**a**) Anomaly percentage of each test video. (**b**) The number of videos in the dataset.

**Figure 2:**
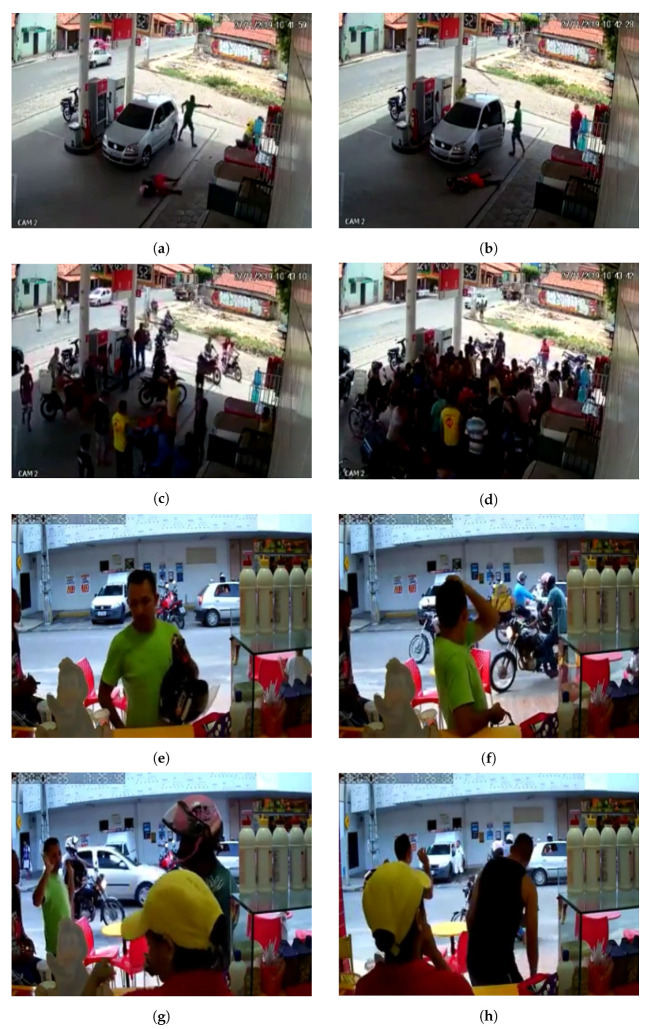
Two samples with clearly different view fields are shown. Four frames from two different videos are illustrated here. The camera used to capture the images from the first video (**a**–**d**) is at a greater height and the people were captured from above. The camera used to capture the images from the second video (**e**–**h**) is at a lower height and the people were captured from the front.

**Figure 3:**
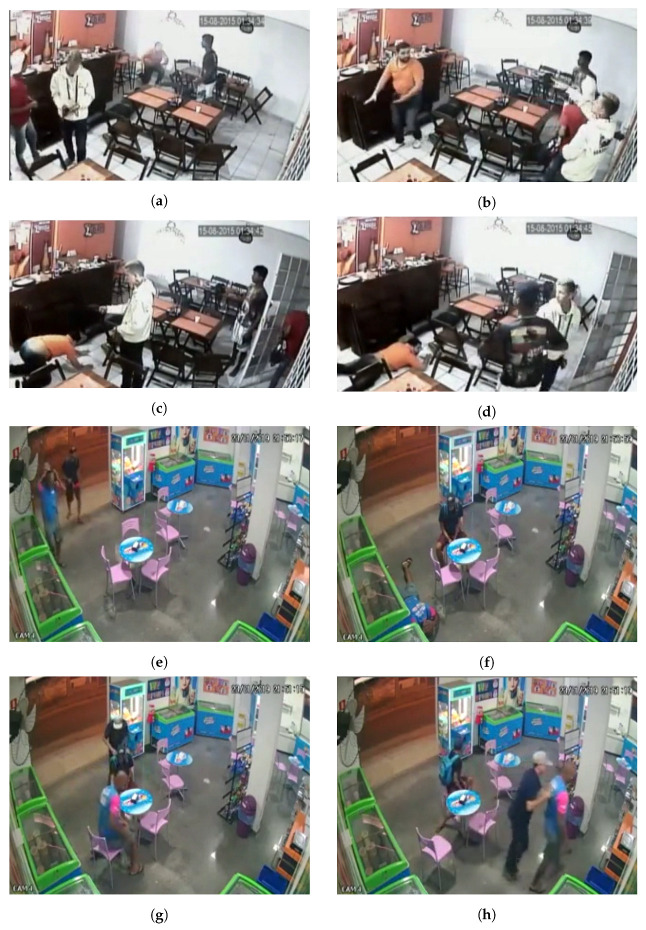
Samples in the abnormal category. Four frames from two different videos are illustrated here. Several image elements can be used to detect a robbery. In the first video (**a**–**d**), the robbery is indicated by the man crouching in the first (**a**) and last (**d**) frames. In the second video (**e**–**h**), the robbery is indicated by the man raising his hands in the first frame (**e**).

All the videos were adjusted to 320 × 240 px and 30 fps. Figure 1a shows the anomaly proportion in each test video, i.e., the percentage of anomaly frames in each video. As described previously, the training videos have no frame–level annotations. Figure 1b shows a video–duration histogram of the dataset. Originally created by Sultani et al. [1], Table 1 provides some general information concerning other similar datasets. Finally, Figure 2 and Figure 3 show some frame samples in the abnormal set to present the general format of a robbery. Note that even though this dataset is focused on only one type of anomaly (robbery), this anomaly is variable enough to make it hard to collect and label a representative dataset in order to train a fully supervised robbery detector.

As shown in Figure 2, in the real world, surveillance cameras can provide different points of view, which is evident when one compares the camera position in the sample shown in Figure 2a–d to the camera position in the sample shown in Figure 2e–h. The camera in the former sample captured the images at a lower height than the camera in the latter sample. Each sample in the dataset was constructed by a camera fixed in a specific position, which makes the dataset representative of the real world.

Furthermore, as shown in Figure 3, there is a large number of different features that can indicate a robbery, e.g., (i) the man with an orange shirt who is lying down in Figure 3c,d, (ii) the interaction of the robber and the victim in Figure 3g,h, (iii) the man with his hands in the air in Figure 3e, and (iv) the man wielding a gun in Figure 2a. One can conclude that the features that compose a robbery situation have a high variability and may also have a large temporal window.

### 3.3. Feature Extractor

The values of the area under the curve (AUC) from the receiver operating characteristic (ROC) curve for the CamNuvem dataset were obtained using the video anomaly detection methods RTFM [2], WSAL [3], and RADS [1]. These methods were chosen due to their high performance in terms of the AUC when they were applied to the UCF–Crime dataset. These techniques expect pre–extracted video features, which must be obtained by applying some feature extraction method to the raw video frames. There are various methods that can be used for this task, but in this work, I3D was used [34]. This design choice is explained below.

Traditional convolutional blocks, when applied to a single image, can aggregate features due to their hierarchical nature. Thus, a CNN can predict some value in the final layer by taking into account features and objects that are in the same image. However, a video may be seen as a set of images (frames) that are linked together in a well-defined order; i.e., each frame is linked to the posterior and previous frames, and information flows through these frames along the temporal axis. When a CNN is applied to a video dataset to extract information, the traditional convolution block can see only a single frame; it ignores the features in the neighbouring frames, and, therefore, on the temporal axis, which can represent an important information loss.

A natural extension that allows the traditional convolution block to deal with temporal datasets (such as video datasets) involves adding a dimension to the convolutional kernels. This technique is known as convolution 3D (C3D) [42]. In this technique, a single convolutional kernel will output a weighted sum of the pixels of a neighbouring frame set, while traditional convolution outputs a weighted sum of the pixels of only one frame, which represents only one moment on the temporal axis. Thus, C3D can be thought of as a convolutional block that takes into account features and information across the temporal axis (when dealing with video datasets).

However, convolution 3D aims to acquire local and temporal features from datasets, and long–range features are aggregated together in the hierarchical neural network structure. This hierarchical structure has a negative impact on computational optimisation because the signals captured by single kernels represent information from a small patch of the image, and the only way to capture long–range dependencies between features is to feed this signal to the deepest layers repeatedly, causing computational inefficiency due to parallelisation constraints. This scenario also occurs in recurrent neural networks (RNNs), which are often utilised for textual datasets; long–range features have to be repeatedly passed through the forward layers to be aggregated together, causing computational difficulties and limiting the power of an artificial neural network.

To confront this problem, non–local neural networks [34], which were inspired by the classical non–local means operator [43], were proposed; they include a neural network block that can improve the computational parallelisation. In this method, the response of a non–local block is a weighted sum of all positions in the input feature map, unlike in convolutional blocks, where the response is computed as a weighted sum of a small local patch of the input feature map. Thus, the video anomaly detection methods (RTFM, WSAL, and RADS) used in this work receive pre–extracted features from raw videos that were extracted by the I3D ResNet Nonlocal model (I3D) [34].

### 3.4. Robust Temporal Feature Magnitude Learning (RTFM)

Consider a set of weakly labelled training videos $D={\left(F_{i},y_{i}\right)}_{i=1}^{\left|D\right|}$ in which $F_{i}$ is a matrix $F\in F\subset R^{T\times D}$ containing the segment feature vectors extracted by some CNN (i.e., I3D [34]), where *T* is the number of segments into which a video is split (all videos are split into 30 segments) and *D* is the dimension of the feature vector. Each $F_{i}$ has a label $y_{i}\in \left\{0,1\right\}$, where $y_{i}=0$ indicates the presence of only normal segments in $F_{i}$, and $y_{i}=1$ indicates the presence of at least one anomaly segment in $F_{i}$. Given a set of video segments *F*, the RTFM method predicts whether each segment is anomalous or not using the model $f_{\phi }\left(s_{\theta }\left(F\right)\right)$, where $s_{\theta }:F\to \chi$ is a temporal feature extractor, with $\chi \in R^{T\times D}$, and $f_{\phi }:\chi \to {\left[0,1\right]}^{T}$ is the segment classifier. The loss function used to minimise the parameters θ and ϕ is

(1) $$\min_{\theta ,\phi }\sum_{i,j=1}^{\left|D\right|}l_{s}\left(s_{\theta }\left(F_{i}\right),s_{\theta }\left(F_{j}\right),y_{i},y_{j}\right)+l_{f}\left(f_{\phi }\left(s_{\theta }\left(F_{i}\right)\right),y_{i}\right),$$

where $s_{\theta }$ is implemented using a pyramid of dilated convolutions [35] over the time domain to capture dependencies from neighbouring segments. Additionally, a temporal self–attention module [34] is used to help aggregate information over all the video segments. $f_{\phi }$ is a fully connected neural network with 3 layers that determines whether the segments represent an anomaly or not. $l_{s}$ is a loss function that aims to minimise the *k* largest segment feature magnitudes from the normal videos and maximise the *k* largest segment feature magnitudes from abnormal videos:

(2) $$l_{s}\left(s_{\theta }\left(F_{i}\right),s_{\theta }\left(F_{j}\right),y_{i},y_{j}\right)=\left\{\begin{array}{cc} max(0,m-d_{\theta ,k}\left(X_{i},X_{j}\right)), & \mathrm{if}\;y_{i}=1,\;y_{j}=0,\\ 0, & \mathrm{otherwise}, \end{array}\right.$$

where *m* is a predefined margin and $X_{i}=s_{\theta }\left(F_{i}\right)$ is the abnormal temporal feature extracted from video *i* ($X_{j}$ is similarly defined). $d_{\theta ,k}\left(X_{i},X_{j}\right))$ is given by

(3) $$d_{\theta ,k}\left(X^{+},X^{-}\right))=g_{\theta ,k}\left(X^{+}\right)-g_{\theta ,k}\left(X^{-}\right),$$

and $g_{\theta },k$ is given by

(4) $$g_{\theta ,k}\left(X\right)=\max_{\Omega_{k}\left(X\right)\subseteq {\left\{x_{t}\right\}}_{t=1}^{T}}\frac{1}{k}\sum_{x_{t}\in \Omega_{k}\left(X\right)}\left|\right|x_{t}{\left|\right|}_{2},$$

where the temporal feature vectors *X* can also be denoted by $X=(x_{1},x_{2},\ldots ,x_{T})$, and $\Omega_{k}\left(X\right)$ represents the set containing *k* temporal feature vectors from *X*, so $|\Omega_{k}\left(X\right)|=k$. The main assumption of this work is that the video segment feature vectors can be classified using their magnitudes ($\ell_{2}$ norm) instead of their spatial separation. This method assumes that the feature vector magnitudes from the normal video are smaller than those from the abnormal videos. Thus, in Equation (4), the mean of the *k* largest feature vector magnitudes from video *X* is calculated; the subtraction of Equation (3) guarantees that the mean magnitude of the abnormal videos ($X^{+}$) is as large as possible, while the mean magnitude of the normal videos ($X^{-}$) is as small as possible. θ in Equations (3) and (4) indicates that in the training process, the feature extractor $s_{\theta }$ will be optimised to produce feature vectors that satisfy this main assumption.

### 3.5. Weakly Supervised Anomaly Localisation (WSAL)

Still considering what was defined in the previous subsection, in a set of weakly labelled training videos $D={\left(F_{i},y_{i}\right)}_{i=1}^{\left|D\right|}$, each video segment in *F* has a feature vector extracted from some CNN. These feature vectors can be denoted by $F=(f_{1},f_{2},\ldots ,f_{T})$. The WSAL method uses Equation (5) to define whether each segment is anomalous or not:

(5) $$S\left(F\right)=\max_{j,k=1,\ldots ,T}g\left(\psi \left(f_{j-d},..,f_{j},\ldots ,f_{j+d}\right),\psi \left(f_{k-d},..,f_{k},\ldots ,f_{k+d}\right)\right),$$

where ψ is a DNN–based architecture called high–order context encoding that aims to extract temporal and spatial information from video frames; it receives an anchored video segment *f* and its 2d neighbours to detect temporal features in a window [−d,d]. *g* is a function that measures the anomaly score margin between the segments $f_{j}$ and $f_{k}$. The max function is used to obtain the two segments with a greater difference in terms of the abnormality score, taking into account the whole video (i.e., all values of *j* and *k*). For the normal videos, it is intended that all segments should present a ψ value near 0, resulting in a *g* value near 0. In the abnormal videos, it is intended that the *g* value should be higher because the anomalous segment will result in a high ψ value. Thus, the result of S(F) is intended to be high when *F* is anomalous (y=1) and low when *F* is normal (y=0). Thereby, one can understand S(F) as a function that computes the anomalous score of a video *F*. Thus, the base loss function used to guide the optimisation is given by

(6) $$ℑ\left(F\right)=max\{0,1-\frac{1}{n_{1}}\sum_{i=1}^{n}\left[S\left(F\right)|y=1\right]+\frac{1}{n_{0}}\sum_{j=0}^{n}\left[S\left(F\right)|y=0\right]\},$$

where $n_{1}$ and $n_{0}$ represent the total number of anomaly samples and the total number of normal samples, respectively.

The high–order context encoding (HCE) module uses two components to mine variations from video features: (i) the semantic component and (ii) the variation component. For the semantic component, it is assumed that the videos can be interpreted as a time series, and the extraction of high–level features can be performed as a regression task. In this way, given feature vectors $\left(f_{1},f_{2},\ldots ,f_{T}\right)$ extracted from consecutive segments, the regression approach can be formulated as follows:

(7) $$\tilde{f_{t}}=\sum_{j=-d,\ldots ,d,d\neq 0}W_{d}f_{t+d}+W_{0}f_{t}+b,$$

where $W_{d}$ is a projection function in the d–th segment and *b* is a bias term. The idea is to obtain discriminative information using a window of 2d segments related to the t–th segment, capturing immediate spatial semantics and local dynamic variations [2]. Then, the WSAL method uses a fully connected network to obtain the final semantic anomaly score for segment *t*, which can be expressed as follows:

(8) $$\psi^{sem}\left(\tilde{f_{t}}\right)=\sigma \left(w_{sem}\tilde{f_{t}}+b_{sem}\right),$$

where $w_{sem}$ and $b_{sem}$ are the weights and biases of the fully connected network. The $\psi^{sem}\left(\tilde{f_{t}}\right)$ network can be stacked so that it has several layers, but it was found that just one layer performed well.

The variation component pays attention to the variation from two adjacent segments, opposing the semantic component, which pays attention to the variation from a window of *d* segments. To do this, the variation component uses the metric $1-cos({\tilde{f}}_{t-1},{\tilde{f}}_{t})$, which results in large values for dramatic changes in segments, where $cos({\tilde{f}}_{t-1},{\tilde{f}}_{t})$ is given by

(9) $$cos\left({\tilde{f}}_{t-1},\tilde{f_{t}}\right)={\tilde{f}}_{t-1}^{⊤}\tilde{f_{t}}/\left({∥{\tilde{f}}_{t-1}∥}^{2}{∥{\tilde{f}}_{t}∥}^{2}\right).$$

Then, the following second–order discrepancy is calculated to obtain the anomaly score:

(10) $$\psi^{var}\left({\tilde{f}}_{t}\right)=\left(2-cos\left({\tilde{f}}_{t-1},\tilde{f_{t}}\right)-cos\left({\tilde{f}}_{t},\tilde{f_{t+1}}\right)\right)/4,$$

where the division by four normalises the anomaly score so that it falls within the range [0,1].

The two semantic components and variation components give context cues that can be used to detect abnormalities in video segments. The L1–distance is used to measure the variation from consecutive segments:

(11) $$S^{sem}\left(F\right)=\max_{i,j=1,..,T}|\psi^{sem}\left({\tilde{f}}_{i}\right))-\psi^{sem}\left({\tilde{f}}_{j}\right))|,$$

(12) $$S^{var}\left(F\right)=\max_{i,j=1,..,T}|\psi^{var}\left({\tilde{f}}_{i}\right))-\psi^{var}\left({\tilde{f}}_{j}\right))|.$$

Putting $S^{sem}$ and $S^{var}$ into Equation (6) results in two new margin losses, $S^{sem}$ and $S^{var}$, respectively. Thus, the final loss function, which takes into account the semantic component and variation component, is given by

(13) $$S^{0}=S^{sem}\left(F\right)+S^{var}\left(F\right)+\frac{\beta }{n}\sum_{i=1}^{n}\sum_{t=1}^{m}\left(\right|s_{t}^{sem}\left|+s_{t}^{var}\right),$$

where β is a sparsity constraint, and $s_{t}^{sem}$ and $s_{t}^{var}$ are the anomaly scores given by Equations (11) and (12), respectively. An approach for dealing with simulated noise data for data augmentation was also proposed, but the formulation is not within the scope of this work. Mathematical proofs, theoretical motivations, and further equation derivations can be found in [3].

### 3.6. RADS

Given a set of weakly labelled training videos $D={\left(F_{i},y_{i}\right)}_{i=1}^{\left|D\right|}$, each video segment in $F_{i}$ has a feature vector extracted from some CNN, given by $F=(f_{1},f_{2},\ldots ,f_{T})$. Given a set of features $F_{a}$ extracted from an anomaly video (y=1) and a set of features $F_{b}$ extracted from a normal video (y=0), the RADS objective function is given by

(14) $$l\left(F_{a},F_{b}\right)=max\left(0,1-\max_{i\in F_{a}}\xi \left(f_{i}\right)+\max_{i\in F_{b}}\xi \left(f_{i}\right)\right),$$

where ξ is a 3–layer fully connected network that maps the feature vector $f_{i}$ to an anomaly score. In this equation, the higher–scoring segment in $F_{a}$ has to be as close as possible to 0, while the higher–scoring segment in $F_{b}$ has to be as large as possible, satisfying the assumption of multiple instance learning, which is explained in Section 3.1. The main disadvantage of this approach in comparison to RTFM (Section 3.4) and WSAL (Section 3.5) is that it does not consider the temporal axis to calculate the anomaly scores of video segments.

### 3.7. Experiments

The WSAL [3], RTFM [2], and RADS [1] methods were used as a baseline for the new dataset introduced in this work. The methods have been previously tested on the UCF–Crime dataset, but in the official RTFM implementation, the four 224 × 224 px corners and central crop, plus their horizontal flipped versions, were extracted from each video frame, resulting in 10 independent images. In the training and test phases, these 10 images are given to the RTFM anomaly detection model and 10 individual anomaly scores are acquired. However, the mean of these 10 independent anomaly scores is calculated to determine whether the original video is an anomaly video or not. Note that this procedure is different from the traditional data augmentation approach, which would result in 10 individual error corrections in the training phase. This procedure will be called 10–crop in the remainder of this work. The original implementations of WSAL and RADS do not perform 10–crop, making a fair comparison of them difficult. Thus, this work created new versions of WSAL and RADS to allow 10–crop samples to be used as input, following the same procedure as RTFM. Likewise, this work also implemented a new version of RTFM to allow non–10–crop samples to be used as input. Thus, a fair comparison of the results of these methods can be performed. The CamNuvem dataset was used in these experiments.

As explained previously, the features were extracted using the I3D model, which was pre–trained on the Kinetics dataset [44]. To verify the influence of the 10–crop method, two versions of the video features were extracted: (i) the first was extracted using the entire video frame, and (ii) the second was extracted using the 10–crop methodology. To avoid frame redundancy, the feature vector was created using sets of 16 consecutive frames, and each feature vector had a size of 2048. All the experiments were performed on a machine with 32 GB of memory and an NVIDIA 1660 Super graphical processing unit with 6 GB of memory.

## 4. Results

In Table 2, the values of the AUC ROC obtained for the RTFM, WSAL, and RADS methods using the CamNuvem dataset with the I3D feature extractor are presented. The values are divided into ‘No 10–crop’ and ‘10–crop’ categories. Moreover, for the CamNuvem dataset (Table 2), the analysis was performed using the entire test partition of the dataset (entire test set row) and using only the abnormal videos in the test partition (only the abnormal videos in the test set row). This division was made because even in an anomaly video, there are normal frames, and because there are many more normal video frames than abnormal video frames in the dataset, even a bad classifier can appear to perform well if it classifies every segment as normal; this can obscure the true performance of the classifier when only the AUC ROC value is considered. To improve the understanding of the capabilities of these methods, the AUC ROC values were also evaluated for only the abnormal videos in the dataset. These values can be seen in the last row of Table 2.

Note that in Table 2, considering the entire test set, the RTFM method obtained an AUC value of 78.76% for the ‘no 10–crop’ dataset and an AUC value of 88.75% for the 10–crop dataset. These values suggest that the 10–crop data augmentation methodology increases the quality of the classification. However, the AUC values decrease for only the abnormal videos in the test set in Table 2; the AUC values decrease to 56.03% and 66.65% for the ‘no 10–crop’ and 10–crop datasets, respectively. These AUC values decrease by 22.73% and 19.40%, respectively. These values suggest that the RTFM method does not perform well when it has to localise the anomaly in an abnormal video. For the WSAL results shown in Table 2, this pattern repeats: the AUC values for the entire test set were 82.01% and 83.45% for the ‘no 10–crop’ and 10–crop datasets, respectively, and these values drop for only the abnormal videos in the test set to 55.22% and 54.59%, decreasing by 26.79% and 28.86%, respectively. Note that the increase in the AUC value for the 10–crop dataset was only 1.44%, unlike the RTFM method, which achieved an improvement of 9.99%. In the RADS results shown in Table 2, the pattern is similar; the AUC values for the entire test set were 79.08% and 82.48% for the ‘no 10–crop’ and 10–crop datasets, respectively, and these values drop for only the abnormal videos in the test set to 51.68% and 50.51%, decreasing by 27.4% and 31.97%, respectively.

**Figure 4:**
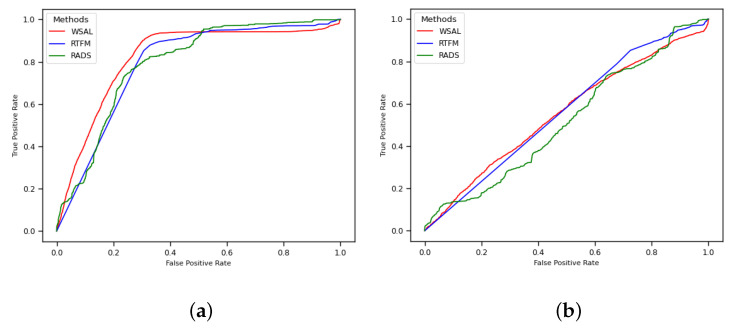
RTFM, WSAL, and RADS ROC curves without 10–crop for CamNuvem dataset. (**a**) Entire test set. (**b**) Only the anomaly videos.

**Figure 5:**
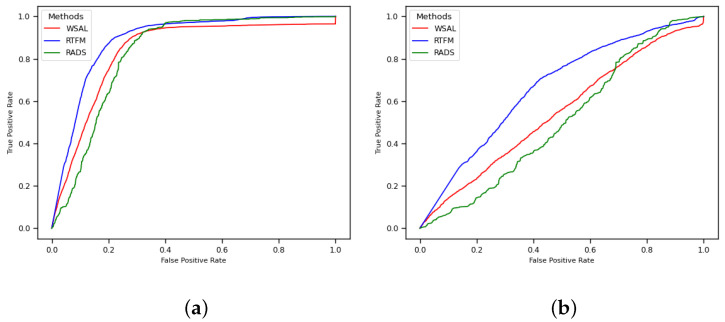
RTFM, WSAL, and RADS ROC curves with 10–crop for CamNuvem dataset. (**a**) Entire test set. (**b**) Only the anomaly videos.

The values shown in Table 2 are illustrated in Figure 4 and Figure 5. Figure 4 depicts the RTFM (blue line), WSAL (red line), and RADS (green line) ROC curves for the entire test set (Figure 4a) and for only the abnormal videos in the test set (Figure 4b) for the ‘no 10–crop’ version of the dataset. Note in Figure 4b that the three methods perform almost randomly when they are evaluated using only anomaly videos, which indicates that further research into robbery anomaly detection and localisation using surveillance videos is required. Likewise, Figure 5 depicts the RTFM (blue line), WSAL (red line), and RADS (green line) ROC curves for the entire test set (Figure 5a) and for only the abnormal videos in the test set (Figure 5b) for the 10–crop version of the dataset. Note that in Figure 5b, the three methods perform almost randomly, but the RTFM method (blue line) achieves a subtle improvement in the performance.

In Figure 4b and Figure 5b, the decrease in the AUC value when the methods are evaluated only on the anomaly videos is due to their inability to localise the anomalies in the videos. When the entire test set is used (Figure 4a and Figure 5a), what is being measured is the ability of the method to identify whether or not a video contains an anomaly. When only the anomaly videos are used, what is being measured is how well the methods can predict where the anomalies are in the video. Thus, what the experiments show is that all of the anomaly detector methods performed poorly when it came to finding the exact positions of the anomalies.

Taking into account the analysis of the entire test set and only the abnormal videos in the test set, the AUC values improved when the 10–crop version of the dataset was used. However, each crop is considered an individual image, so this version requires more computational power.

## 5. Conclusions

This work presented the CamNuvem dataset, a new weakly labelled dataset containing real–world surveillance videos showing robberies. The previous sections discussed the importance and innovation of this dataset, which will enable the development of human behaviour detection systems that can be used to detect robberies in real life. An initial benchmark was also constructed to allow a comparison of the results with future studies.

The experimental results showed that the feature extraction technique strongly influences the results. Furthermore, it was also shown that the methods are not good at localising anomalies in videos. Thus, future work will involve (a) proposals to extract features based on human interaction in videos and (b) the investigation of methods that can be used to achieve better anomaly localisation. Thus, one can conclude that video surveillance anomaly detection has many research opportunities; new approaches are needed to make real–world applications possible.

## Figures and Tables

**Table 1:** Comparison of several video surveillance anomaly detection datasets [1]. * Values in parentheses refer to the novel robbery samples only.

	Number of Videos	Average Number of Frames	Dataset Length	Anomaly Examples
Avenue [37]	37	839	30 min	Bikers, small carts, walking across walkways
BOSS [38]	12	4052	27 min	Bikers, small carts, walking across walkways
Subway Entrance [39]	1	121,749	1.5 h	Wrong direction, no payment
Subway Exit [39]	1	64,901	1.5 h	Wrong direction, no payment
UCSD Ped1 [36]	70	201	5 min	Run, throw, new object
UCSD Ped2 [36]	28	163	5 min	Run
UMN [40]	5	1290	5 min	Harass, disease, panic
UCF–Crime [1]	1900	7247	128 h	Abuse, arrest, arson, assault, accident, burglary, fighting, robbery
UCF–Crime Extension [41]	240	3060	7.5 h	Protest, violence using Molotov cocktails, fighting
Ours	972 (486) *	6329 (2303) *	57 (10.37) * hours	Robbery

**Table 2:** AUC ROC values of the anomaly detection methods RTFM, WSAL, and RADS, which were used to detect abnormalities in the CamNuvem dataset. The features were extracted by I3D.

RTFM		WSAL		RADS	
No 10–crop	10–crop	No 10–crop	10–crop	No 10–crop	10–crop
Entire test set					
78.76%	88.75%	82.01%	83.45%	79.08%	82.48%
Only the abnormal videos in the test set					
56.03%	66.35%	55.22%	54.59%	51.68%	50.51%

## Data Availability

The data presented in this study are openly available at https://drive.google.com/drive/folders/1LA5OYMXG8rmymJaZ-Moji6Ke6YNGuAyF?usp=sharing (accessed on 23 October 2022).

## Abbreviations

The following abbreviations are used in this manuscript:

DNN	Deep Neural Network
GAN	Generative Adversarial Network
CNN	Convolutional Neural Network
RNN	Recurrent Neural Network
LSTM	Long–Short Term Memory
HOG	Histogram of Oriented Gradients
AUC	Area Under Curve
ROC	Receiver Operating Characteristic
RTFM	Robust Temporal Feature Magnitude Learning
WSAL	Weakly Supervised Anomaly Localisation
MIL	Multiple Instance Learning
